# What Advanced Practitioners Need to Know About the Diagnosis and Treatment of Patients With Pancreatic Cancer

**DOI:** 10.6004/jadpro.2017.8.3.7

**Published:** 2017-04-01

**Authors:** Joseph M. Herman, Amy Hacker-Prietz

**Affiliations:** 1 The University of Texas MD Anderson Cancer Center, Houston, Texas;; 2 Johns Hopkins University, Baltimore, Maryland

## Abstract

Multidisciplinary clinics for pancreatic cancer such as one established at Johns Hopkins offer advantages in the care of patients with pancreatic cancer. Advanced practitioners can play a role in multidisciplinary care, providing symptom management and acute care, and clinical trial enrollment/consent process.

Progress in the field of oncology in general has failed to produce a major impact on prognosis in pancreatic cancer, which continues to have one of the highest fatality rates. Reasons for the persistently poor prognosis of pancreatic cancer are multifaceted, as Joseph M. Herman, MD, of The University of Texas MD Anderson Cancer Center in Houston, and Amy Hacker-Prietz, PA-C, of Johns Hopkins University in Baltimore, explained during a presentation at JADPRO Live 2016.

## PROGNOSTIC FACTORS

The speakers first described the multiple factors leading to poor prognosis of pancreatic cancer, including the organ’s proximity to critical blood vessels, an aggressive biology that leads to early metastatic spread, and physiologic factors that contribute to poor tolerance to treatment, such as exocrine insufficiency and cachexia.

Since therapeutic options for pancreatic cancer remain limited, the disease can progress quickly to a treatment-resistant state. Delayed diagnosis and treatment contribute substantially to the poor prognosis, and little progress has been made toward identifying biomarkers that would allow for earlier diagnosis and treatment, Dr. Herman and Ms. Hacker-Prietz noted.

Still another contributing factor to the poor prognosis in pancreatic cancer is that many patients do not receive evidence-based or guideline-supported care.

"Unfortunately, there are not many therapies we can give our patients," said Dr. Herman. "That makes our job difficult. You want to provide hope, but you also want to be realistic about expectations."

As with other poor-prognosis cancers, the epidemiology of pancreatic cancer reflects the reality as well as any clinical or pathologic factors. Almost 50,000 new cases occur each year, and the number of cases continues to increase with the aging of the Baby Boomer population. The annual mortality is 41,000, making pancreatic cancer the third leading cause of cancer death in the United States (Khorana et al., 2016).

The vast majority of patients have no established risk factors. Notable exceptions include individuals with a family history of *BRCA* mutations or cancers associated with the mutations. Other uncommon inherited conditions also predispose people to an increased risk of pancreatic cancer: familial atypical multiple mole melanoma; familial pancreatitis; and Peutz-Jeghers syndrome.

## STAGING

Only 15% to 20% of patients have potentially resectable disease at diagnosis. Negative surgical margins offer the only potential cure for pancreatic cancer. Patients with complete, incomplete, or margin-positive resection have progressively decreasing survival (Conlon, Klimstra, & Brennan, 1996). Accurate staging is crucial to avoid nonbeneficial surgery, because margin-positive surgery has a prognosis similar to that of metastatic disease.

"One of the most important messages to take away from this presentation is that staging is paramount," said Dr. Herman. "Patients will perseverate on ’What stage am I? Where do I fit in this whole picture? Am I metastatic? Am I localized?’ That’s what they’re going to look at online, and as they go through their treatment trajectory, that’s what they’re going to pay attention to. It’s important to establish the staging upfront."

## MULTIDISCIPLINARY CARE

Within the context of a complex disease process associated with a poor prognosis, the value of multidisciplinary care has become an established approach at a growing number of centers. Multidisciplinary clinics offer many potential advantages in the care of patients with pancreatic cancer.

- Specialists work together in a collaborative environment, which helps provide evidence-based care and development of consensus recommendations
- Comprehensive, consensus-based care in a single location addresses and reduces patient anxiety and improves convenience
- Patient access and exposure to support services are enhanced
- Enrollment in clinical trials is facilitated.

"It helps us, as well, to sit in with other providers in oncology and surgery, all at the same time, so we benefit from their education and then move forward and help patients," said Ms. Hacker-Prietz.

Collaborative consultation and assessment can improve the efficiency of care, as well as the quality, they continued. In the traditional care paradigm, a patient meets individually with each provider. Using an example of a patient with new jaundice, 6 weeks or more could pass before each provider could assess the patient, determine the cause of the jaundice, and decide on a course of action.

In contrast, by meeting with a multidisciplinary team, the patient "gets everything in one day, and you can reduce that workup time by about 4 weeks," said Dr. Herman, who until recently worked at Johns Hopkins alongside Ms. Hacker-Prietz. Imaging performed early in the course of a workup is no longer valid 4 weeks later in about 25% of cases, emphasizing the importance of timing on prognosis and outcome in pancreatic cancer.

The multidisciplinary cancer clinic differs substantially from a tumor board, they emphasized, and very different discussions occur. "When you have someone who has the experience of seeing patients upfront, you might have someone say ’I know you are talking about [chemotherapy], but Mr. Jones doesn’t want [chemotherapy]. He wants to go fishing,’ " said Dr. Herman. " ’The last time he came in, he could barely get on the table. Let’s be realistic.’ "

"We need that sounding board…. We encourage people to be vocal and focus on empowering every part of the team. Personally, I value it," added Dr. Herman, "because the worst thing is to do something that is going to hurt someone." At Johns Hopkins, the speakers found that multidisciplinary review led to a change in diagnosis or treatment in 28% of cases (Pawlik et al., 2008).

## ESSENTIALS OF CARE

High-quality imaging and accurate radiologic reporting are essential to the care of patients with pancreatic cancer (Al-Hawary et al., 2014a, 2014b). Every patient should undergo fine-needle aspiration biopsy by endoscopic ultrasound guidance, unless percutaneous access is the only option available (National Comprehensive Cancer Network, 2016; Khorana et al., 2016). Optimal imaging also includes thin-slice, multidetector computed tomography (CT) scanning, which permits three-dimensional reconstruction, plus a CT angiogram. Magnetic resonance imaging is used infrequently in selected circumstances, such as distinguishing between malignant and benign tissue. Collectively, the recommended imaging provides the anatomic information needed for accurate staging.

Beyond the subgroup of patients with resectable disease at diagnosis, about 35% of patients have borderline-resectable disease, 37% have metastatic disease, and 8% have end-stage disease (advanced stage combined with a poor performance status or other high-risk features).

## IMPACT OF RESECTABILITY ON TREATMENT APPROACH

Borderline resectable is a relatively new term that refers to the tumor’s relationship with surrounding vasculature. In simplest terms, "borderline" refers to the extent of tumor involvement with a blood vessel’s circumference, Dr. Herman explained. Borderline resectable means that a tumor has contact with less than 180° of the vessel’s circumference and, ideally, causes little or no deformity in the vessel’s shape. Another consideration is whether a vein or artery is involved. In general, veins can be resected with fewer complications when compared with arteries.

The approach to treatment depends primarily on the resectability of the disease at diagnosis. Patients with resectable tumors almost always undergo surgery, and many receive neoadjuvant chemoradiation or adjuvant therapy. Patients with borderline-resectable disease often receive chemotherapy, with the goal of reducing tumor volume or involvement to improve surgical outcome. If a tumor is borderline resectable but anatomically challenging, a patient may receive chemotherapy with or without radiation therapy. The clinical intent in this case is palliative.

Patients with unresectable disease at diagnosis may receive chemotherapy, chemoradiation therapy, or stereotactic body radiotherapy. The intent here is palliative, regardless of the treatment strategy.

"We have sort of converged in our thinking about treatment of patients with localized, nonmetastatic disease," said Dr. Herman. "They receive maximized chemotherapy and then radiation therapy. We evaluate them every 2 or 3 months and often keep them on maintenance therapy. Unfortunately, we don’t know what the ’right’ treatment is, and so we try to take into account what the patients and their families want."

## TREATMENT OPTIONS AND CHALLENGES

**Chemotherapy**

For years, gemcitabine monotherapy represented the standard of care, even though the treatment resulted in only a modest survival benefit. Recently, two regimens have emerged as the most commonly used chemotherapy standards: FOLFIRINOX (folinic acid/fluorouracil [5-FU]/irinotecan/oxaliplatin) and gemcitabine/nanoparticle albumin-bound (nab)-paclitaxel (Abraxane).

Investigators of a French multicenter, randomized trial compared FOLFIRINOX with gemcitabine monotherapy in 342 patients with metastatic pancreatic cancer (Conroy et al., 2011). Patients treated with the combination regimen had a median overall survival of 11.1 months vs. 6.8 months with gemcitabine and a median progression-free survival of 6.4 vs. 3.3 months (*p* < .001 for both comparisons).

The combination of gemcitabine and nab-paclitaxel was compared against gemcitabine alone in a randomized, multicenter, international, phase III trial involving 861 patients with advanced pancreatic cancer (Von Hoff et al., 2013). Treatment with gemcitabine/nab-paclitaxel led to a median overall survival and progression-free survival of 8.5 and 5.5 months, respectively, as compared with 6.7 and 3.7 months (*p* < .001 for both comparisons).

**Radiation Therapy**

Pancreatic cancer poses some unique challenges in terms of radiation therapy, said Dr. Herman. Because of the proximity of the pancreas to the small bowel, radiation therapy can cause ulceration, bleeding, and bowel perforation. The risk of late bowel complications increases with the radiation dose.

Over the years, advances in radiation therapy technology and techniques have improved the ability to treat pancreatic cancer with less risk to surrounding tissues. The advent of intensity-modulated radiation therapy improved targeting of higher doses of radiation with reduced exposure to surrounding areas. Active breathing control reduced movement of the pancreas to allow better targeting of tumors and treatment margins.

Use of markers (fiducials) to delineate the treatment target facilitated further homing of the radiation to the tumor. Markers gained additional prominence with the emergence of stereotactic body radiation therapy (SBRT), which permitted delivery of a higher dose with greater precision and over a shorter time.

"We can now irradiate these tumors in 5 days instead of 28 to 30 days," said Dr. Herman. "Our patients can come in, get x number of days of irradiation, and then go home. Their quality of life, right then and there, is so much better."

The ultimate goal is to use radiation therapy to "sterilize" tumors and allow patients to avoid surgery altogether in cases when surgery might have a limited impact on outcome, he added. An impact has already been seen in the treatment of patients with borderline-resectable disease. In some centers, targeted radiation therapy has increased the rate of resectability from about 10% to 40% in the subgroup of patients with borderline-resectable tumors.

## GENETIC MARKERS

The next step in the evolution of treatment involves analysis of tumor genetics to select patients for different types of treatment. Preliminary results suggest that the presence of certain genes may provide guidance toward the most appropriate treatment options.

In keeping with the search for useful genetic markers, analysis of conventional clinicopathologic factors has helped identify patients who are likely to benefit from surgery, which still offers patients the only chance of cure. Studies at Johns Hopkins showed that surgery significantly improved survival in patients who received more than one chemotherapeutic agent, who received chemotherapy for at least 4 months, and who received SBRT doses of at least 33 Gy. Johns Hopkins researchers have also asked whether some patients can forgo radiation therapy. Preliminary data suggested that the addition of radiation improved margin outcomes and the rate of pathologic complete response, as compared with chemotherapy alone.

## ROLE OF ADVANCED PRACTITIONERS

The nation’s 333,000 advanced practitioners have had a major impact on health-care delivery in the United States, but their representation in oncology remains limited, according to Ms. Hacker-Prietz, who noted that only 2.3% of the nation’s advanced practitioners currently work in oncology (National Commission on Certification of Physician Assistants, 2015; American Association of Nurse Practitioners, 2015).

Given that more patients with cancer are living longer, advanced practitioners are well suited to address the ongoing needs in direct patient care, including symptom management, procedures of various type and complexity, and prescriptive authority (as allowed by state regulations). Advanced practitioners can work independently, and their services are reimbursable at rates of 85% to 100% of physician payments.

At Johns Hopkins, the gastrointestinal radiation oncology clinical model encompasses a team consisting of an attending physician, PA, nurse, resident, clinical coordinator, administrative assistant, and research staff. Advanced practitioners provide for continuity of care (as residents rotate out of the service every 7 weeks), delivery of direct patient care, and provision of services that allow physicians to attend to other responsibilities, she said. The Table highlights the role of advanced practitioners in gastrointestinal services.

**Table:**
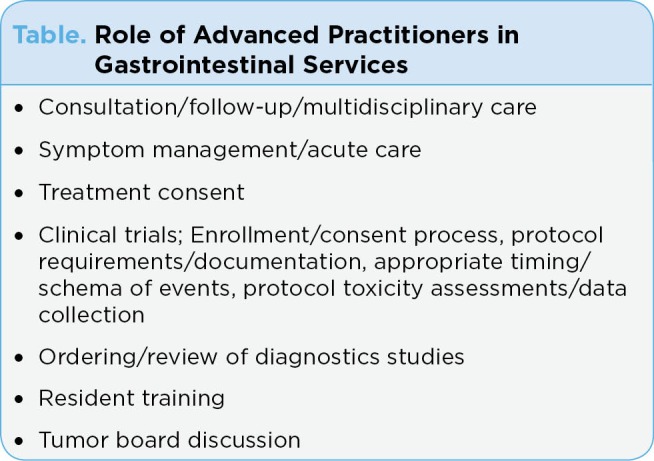
Role of Advanced Practitioners in Gastrointestinal Services

Beyond direct care, advanced practitioners can improve patient care and quality of life by addressing many other issues affecting the patient: goals, place of residence and support system, family planning and fertility for younger patients, symptom management, physical therapy, and work/leisure activities.

Advanced practitioners often are the principal providers of patient education in the oncology setting, which includes symptom management, management of expectations, "flow" of treatment and care, and services available to them (i.e., social work, nutrition, physical therapy, pain management).

"Talk with family members," said Ms. Hacker-Prietz. "They know patients sometimes can be pretty stoic and don’t always express some of the things they’re going through."

## PAIN AND EXOCRINE PANCREATIC INSUFFICIENCY

Advanced practitioners play a key role in managing two types of conditions affecting a majority of patients with pancreatic cancer: pain and exocrine insufficiency.

**Pain**

About 70% of persons with active cancer and 40% of cancer survivors report having pain, and as many as 80% of patients with pancreatic cancer have pain as the initial symptom, said Ms. Hacker-Prietz. The pain affects multiple sites in 75% of cases, and three-fourths of patients require opioids for adequate pain relief. One-third of patients have functional deficits as a result of pain, and in 60% of cases, the pain adversely affects quality of life (Grossman & Nesbit, 2013; Paice et al., 2016).

"Pain is scary to patients. Even if they aren’t experiencing a lot of pain, they are anticipating it," said Ms. Hacker-Prietz.

A good history and physical examination often can define a patient’s pain experience. Clinicians should encourage patients to maintain pain logs. Clinicians should also review patient records to look for factors that can pinpoint the origin, timing, and other factors associated with pain. Ms. Hacker-Prietz advised to take into account special considerations that can influence the pain experience: older age; comorbidities; liver or kidney dysfunction; obstructive sleep apnea; or coronary artery disease.

Pain management should initially focus on the various treatment options, Ms. Hacker-Prietz continued. They include opioid vs. non-opioid medication, interventional procedures, nonpharmacologic therapies, and psychosocial interventions.

"The most important thing is to screen for pain at every visit," she said. "Even though the first time patients come through the clinic they might not have pain, ask about it every time you see them. If you make adjustments to their regimen for pain, continue to ask them, so you can see how well the pain is controlled or whether it has changed."

**Exocrine Pancreatic Insufficiency**

Exocrine pancreatic insufficiency is a common and difficult effect of pancreatic disease. Contributing to this problem are loss of parenchyma, ductal obstruction, and surgery or a surgery-related injury. Potential symptoms are equally varied and can seem nonspecific in many cases: diarrhea, steatorrhea, abdominal distension, weight loss, malabsorption, vitamin B12 deficiency, and exacerbation of existing motility disorders.

Enzyme replacement therapy is standard for patients with pancreatic cancer. Treatment should begin early with appropriate dosing. For some patients, cost may be an issue; this should be taken into consideration, along with the availability of assistance programs.

## MULTIDISCIPLINARY CLINIC FOR PANCREATIC CANCER

Johns Hopkins established a multidisciplinary clinic for pancreatic cancer in 2008, beginning with a handful of patients. Over time, the program has increased and now accommodates as many as 14 patients. The clinical component includes representatives from surgery, medical oncology, radiation oncology, gastroenterology/endoscopy, pathology, radiology, pain and palliative care, and clinical trials/research. Over 1 or 2 days, the clinic meets with patients and reviews the history, images, and pathology. Ms. Hacker-Prietz added that advanced practitioners participate in every aspect of clinic activities.

Beyond interaction with the clinic members and other specialties, advanced practitioners can play a role in community engagement; clinical trials; research; as well as education and mentorship of other advanced practitioners, residents, and medical students.

"Advanced practitioners can assist in improving patient treatment flow, provider work flow, and overall patient care," she said. "Use of advanced practitioners can be cost-effective and time-efficient, particularly in symptom management. We can help fill the deficit resulting from increasing demand for services. In the academic setting, advanced practitioners can contribute to teaching, publications, and clinical trials."
